# Involvement of the Cav3.2 T-type calcium channel in thalamic neuron discharge patterns

**DOI:** 10.1186/1744-8069-7-43

**Published:** 2011-06-04

**Authors:** Yi-Fang Liao, Meng-Li Tsai, Chien-Chang Chen, Chen-Tung Yen

**Affiliations:** 1 Institute of Zoology, National Taiwan University, Roosevelt Road, Taipei, Taiwan; 2 Department of Biomechatronic Engineering, National Ilan University, Ilan, Taiwan; 3 Institute of Biomedical Sciences, Academia Sinica, Academia Road, Taipei, Taiwan

## Abstract

**Background:**

Mice that have defects in their low-threshold T-type calcium channel (T-channel) genes show altered pain behaviors. The changes in the ratio of nociceptive neurons and the burst firing property of reticular thalamic (RT) and ventroposterior (VP) neurons in Cav3.2 knockout (KO) mice were studied to test the involvement of thalamic T-channel and burst firing activity in pain function.

**Results:**

Under pentobarbital or urethane anesthesia, the patterns of tonic and burst firings were recorded in functionally characterized RT and VPL neurons of Cav3.2 KO mice. Many RT neurons were nociceptive (64% under pentobarbital anesthesia and 50% under urethane anesthesia). Compared to their wild-type (WT) controls, fewer nociceptive RT neurons were found in Cav3.2 KO mice. Both nociceptive and tactile RT neurons showed fewer bursts in Cav3.2 KO mice. Within a burst, RT neurons of Cav3.2 KO mice had a lower spike frequency and less-prominent accelerando-decelerando change. In contrast, VP neurons of Cav3.2 KO mice showed a higher ratio of bursts and a higher discharge rate within a burst than those of the WT control. In addition, the long-lasting tonic firing episodes in RT neurons of the Cav3.2 KO had less stereotypic regularity than their counterparts in WT mice.

**Conclusions:**

RT might be important in nociception of the mouse. In addition, we showed an important role of Cav3.2 subtype of T-channel in RT burst firing pattern. The decreased occurrence and slowing of the bursts in RT neurons might cause the increased VP bursts. These changes would be factors contributing to alternation of pain behavior in the Cav3.2 KO mice.

## Background

Thalamic burst and tonic firing patterns are important in many behavioral states [1-3]. In relate to sensory functions, firing patterns are highly correlated to the responsiveness to sensory stimulation [4-6]. Thus, thalamic burst firing is thought to gate sensory inputs. Thalamic burst firing occurred in human subjects after noxious stimulation [7-9] and was also found spontaneously in human neurogenic pain patients [10,11]. Furthermore, suppression of thalamic bursts genetically or pharmacologically changes nociceptive responses. For example, thalamic burst firing was absent in Cav3.1 subtype T-channel knockout (KO) mice, and these mice showed increased visceral nocicepetive writhing behavior [12]. Mice intrathalamically treated with mibefradil (a T-channel blocker) showed increased paw withdrawal to thermal stimulation [12]. Therefore, thalamic burst firing may be important in pain functions.

T-channel is critically important for the generation of thalamic burst firing [13-15]. The T-channel is activated by transient depolarization from a hyperpolarized state. The Ca^2+ ^influx induces a train of high-frequency Na^+ ^spikes, i.e., the burst firing. There are three subtypes of the T-channel, namely, Cav3.1, Cav3.2, and Cav3.3 [13,16,17]. Cav3.1 and Cav3.2 have faster activation and inactivation properties than does Cav3.3 [13,17]. The distribution of these subtypes in the thalamus is nucleus specific [18]. Cav3.1 is only expressed in the ventroposterior nucleus (VP), but Cav3.2 and Cav3.3 are expressed in the reticular thalamic nucleus (RT) [18]. VP neurons usually exhibit 2~7 spikes in a burst, and the mean frequency is in the range of 250~400 Hz [2,3,19-21]. Comparing to VP bursts, those of RT neurons are longer, and spikes within a burst have an accelerando-decelerando characteristic. When RT neurons fire as a burst, they usually begin with several repeated bursts and finish with a long-lasting tonic spiking tail of variable durations [2,22].

Rodent VP has very few interneurons [23-25]. Somatosensory and pain functions of thalamocortical neurons in the VP have been studied intensively. VP thalamocortical neurons receive dense GABAergic inhibitory input from topographically connected RT neurons [26,27]. It is the balance between the excitatory TC neurons and the inhibitory RT neurons that shape the thalamic responses to peripheral stimulation. The nociceptive function of the RT neurons, however, has been less studied [28,29]. Considering the important role of RT and VP in the overall nociceptive pathway, it would be of interest to examine RT and VP neurons simultaneously in their nociceptive responsiveness, and to compare their bursting activity together.

Recent studies showed that specific subtypes of the T-channel have diverse impacts on pain function. Cav3.1 KO mice and mice treated with T-channel blocker in the VP thalamic nuclei showed increased responses to thermal, visceral, and inflammatory painful stimuli [12]. In contrast, Cav3.2 KO and spinal cord Cav3.2 mRNA antisense-treated animals showed decreased responses to mechanical, thermal, visceral, and inflammatory stimuli [30,31]. Only VP firing pattern of the Cav3.1 KO mice have been examined previously [12], therefore, we used multi-channel single-unit recording method to study the burst firing properties of RT and VP neurons in Cav3.2 KO mice in the present report to explore the thalamic contribution to the altered pain responsiveness of this animal.

## Methods

All experimental procedures were approved by the Institutional Animal Care and Use Committee of National Taiwan University and adhered to the guidance established by Codes for Experimental Use of Animals from the Council of Agriculture, Taiwan. Animals were kept in a 12-h dark/light cycle environment at a temperature of 22°C with food and water available ad libitum.

The experiments were carried out on Cav3.2 KO and WT B6/C57 mice weighing 27~30 g. Anesthesia was induced by either sodium pentobarbital (50 mg/kg; i.p) and supplemental diluted anesthetics (10 mg/kg), or urethane (1.4 g/kg; i.p.). The mouse stayed in a state with no flexor reflex to a tail pinch. A feedback-control heating pad was used to maintain the rectal temperature at 37.5 °C. Bilateral craniotomies were performed to expose the electrode penetration area (VP and RT forelimb or hind limb region: P 0.9~1.5 mm, L 1.8~2.5 mm, relative to the bregma, at a depth of 3.5~4.2 mm). The dura mater of this area was removed, and the foramen magnum was opened to avoid brain swelling. A stainless-steel screw was implanted into the skull above the cerebellum as a reference electrode.

### Extracellular recording

**Figure 1:**
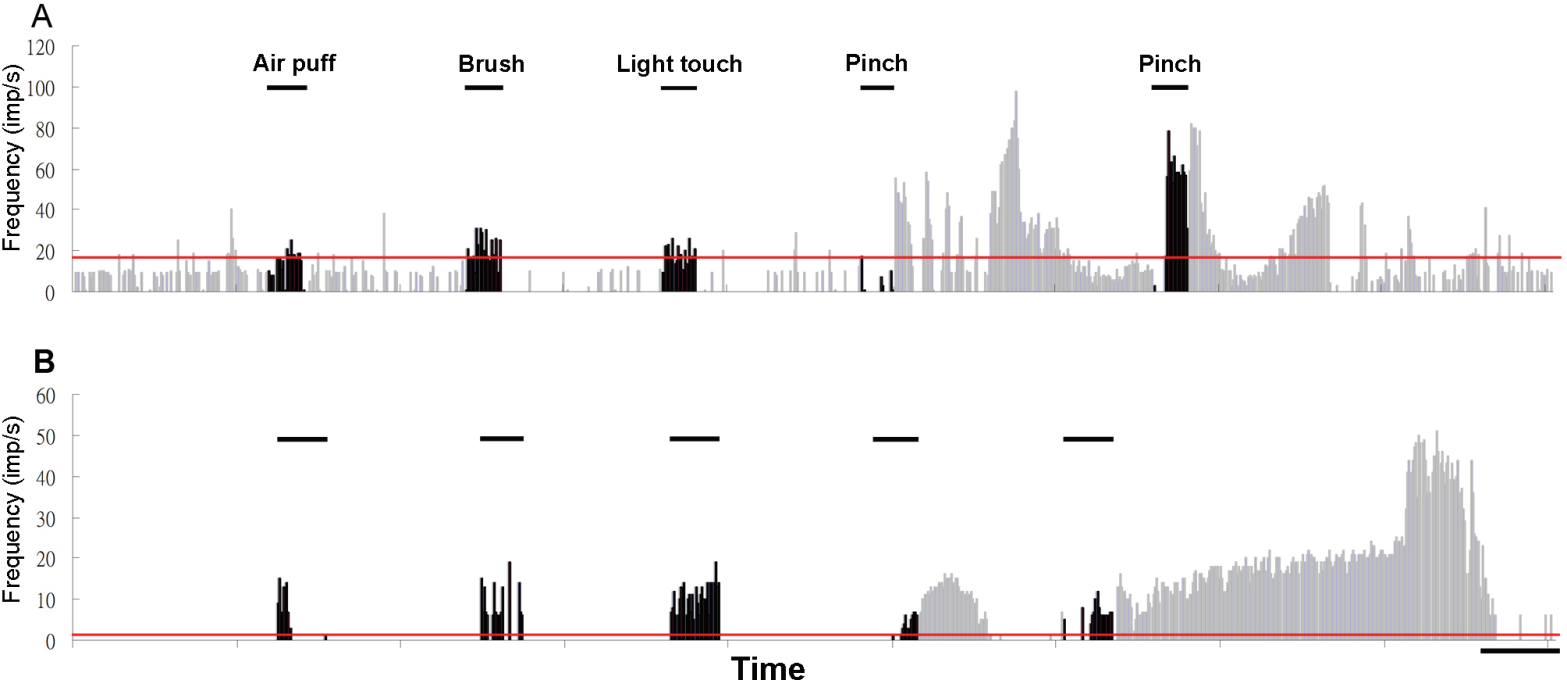
**Examples of nociceptive RT units in a WT (A) and a Cav3.2 KO (B) mice**. Rate histograms show the unit activity changes during innocuous and noxious stimulations (bin = 1 s). The horizontal bars indicate periods for the air puff, brush, light touch and pinch stimulations, respectively, applied to the center of the receptive field of the units, both in the contralateral forepaw. Pinch stimuli repeated twice with the same vessel clamp. Nociceptive units responded to pinching consistently. Light touch units did not respond to pinching. Note both units were inhibited in the beginning of the pinch and followed by excitation and long afterdischages. Red horizontal lines are 95% confidence interval calculated from basal activity. Time scales are 50 s.

Thirty-two-channel Michigan probes (tetrode probe: a4 × 2 tetrode-5 mm 150-200-312; linear probe: a4 × 8-5 mm 50-200-177, NeuroNexus Technologies, Ann Arbor, MI) were used for extracellular recording. Only neurons with a receptive field in either the forelimb or hindlimb were used. When a recording site was selected, spontaneous neuronal activities were first recorded for 20~30 min. This was followed by somatosensory stimulations including air puffs, brush, light tap and pinch (by a small artery clamp). Each stimulation was applied for 20 s with inter-stimulus intervals of 90 s (Figure 1). The responsive units were determined by the histogram bins exceeded mean ± 2.33 calculated from 2 min baseline activities. RT and VP units responded to the pinch stimulation were be defined as nociceptive units; units responded to air puff, brush or light tapping stimuli were light touch (LT) units.

**Figure 2:**
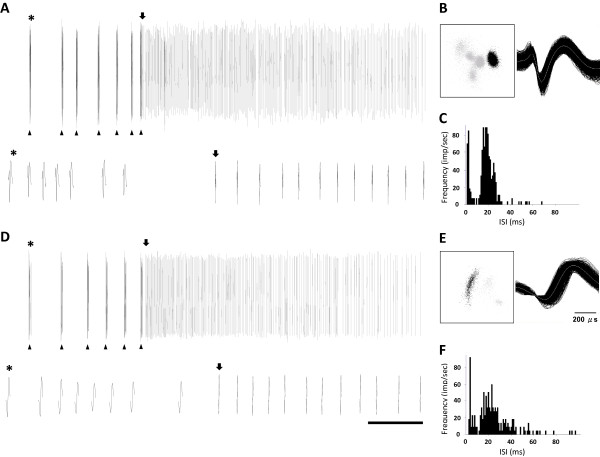
**Examples of spontaneous burst and tonic activity of RT units in a WT (A, B, and C) and a Cav3.2 KO (D, E, and F) mice**. (A and D) Top panel: an 8-s-long trace of the spontaneous activity of the RT unit. The arrowheads at the bottom of the beginning of the trace indicate burst firing. The parts pointed to by the stars and arrows are segments expanded in the lower panels. (B and E) PC1-PC2 plot (left panel) and superimposed wavelet plot (right panel) of the spike clusters of the units shown in figures A and D. (C and F) Inter-spike-interval (ISI) histograms of the examples in A and D; bin = 1 ms. Time scales for the upper panels of A and D are 1 s and for the expanded traces are 10 ms for the burst and 0.1 s for the tonic activities.

**Figure 3:**
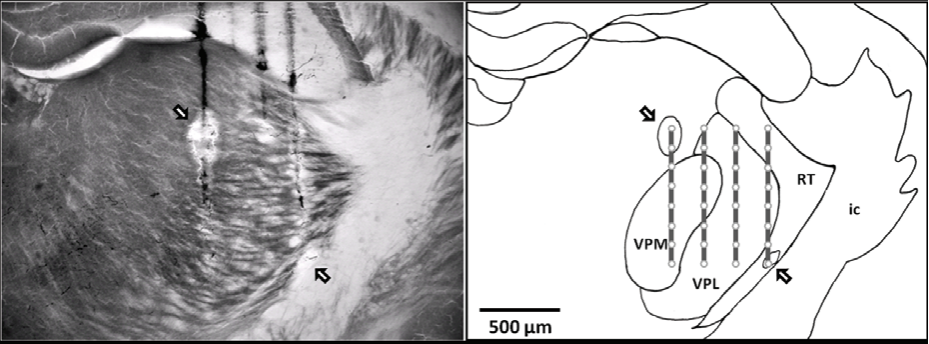
**Identification of recording sites**. Photomicrograph of a brain section counterstained with HRP-DAB and cytochrome oxidase to highlight the penetration tracks and to aid subnuclei identification (Left panel). In the right panel is the Camera Lucida drawing of the same section with reconstruction of the recording sites. The vertical dark gray lines represent the four electrode tracts and open circles represent the 32 recording sites of the multi-channel Michigan probe. The arrows point to the lesion marks, one in the top left and the other in the bottom right corners of the 4-array recording electrode. ic, internal capscle; RT, reticular thalamic nucleus; VPL, ventroposterior lateral nucleus; and VPM, ventroposterior medial nucleus.

The next recording site was separated by at least 500 μm in the same recording track. In most of the experiments, only one recording penetration was made on each side of the brain. Thalamic unit activities were recorded with a multi-channel acquisition processor system (MAP, Plexon, Dallas, TX). Wavelets were stored and further analyzed using Offline-sorter (Plexon), NeuroExplorer (Nex Technologies, Littleton, MA), and SigmaPlot software (Systat Software, San Jose, CA). Only units which met the following three criteria were included for further data analysis: (1) had a receptive field in either the forelimb or hindlimb (Figure 1); (2) single units with well-isolated waveforms (Figure 2B and 2E, right panels) and clusters (Figure 2B and 2E, left panels); and (3) the recording sites could be located in RT or VP by histological verification (Figure 3).

To determine the precise positions of the recording sites, the recording probes were dipped in a horseradish peroxidase (HRP) solution before being inserted into the brain, and at the end of the recording session, small positive DC currents of 5~10 μA in intensity were applied for 30 s to two selected points in the recording arrays. After the experiments, animals were deeply anesthetized with sodium pentobarbital (65 mg/kg) and perfused with 15 ml saline (NaCl 0.9%), followed by 25 ml 4% formaldehyde. The brains were removed and placed in a mixed solution of 30% sucrose and 4% formaldehyde for post-fixation for 24 h. Serial brain sections, 100 μm thick, were cut on a frozen microtome, and tissues were stained with HRP-DAB to identify the penetration track. Finally, cytochrome oxidase staining was used to counterstain and demarcate the thalamic nuclei.

### Burst firing analysis

To compare burst patterns, we used the burst analysis function in the NeuroExplorer software. The following burst criteria were used: a maximum interval to start a burst of 10 ms, a maximum interval to end a burst of 10 ms, a minimum inter-burst interval of 100 ms, a minimum burst duration of 4 ms, and a minimum number of spikes in a burst of 3 or 4 [2]. To compare the accelerando-decelerando pattern, ISIs of the first burst in each and every long train of bursts were analyzed. Tonic firing was also analyzed in WT and Cav3.2 KO RT neurons. In RT neurons, the long-lasting tonic spike trains followed several initial burst firings (Figure 2A, D). This firing characteristic presented two prominent peaks in the ISI analysis (Figure 2C, F). In this analysis, all of the RT units were calculated over 20~30 min of spontaneous activities (bin = 1 s), and the single spike train behind the bursts was extracted to further analyze the tonic firing duration, and the mean frequency, spikes, and ISI distribution within a tonic firing.

### Tonic firing analysis

Considering that tonic firing had an uneven length, we used a normalized length for each tonic episode. The normalized duration of each episode was 100, and the normalized timing of spike firing was calculated as the percentage of the ISI to the total duration of the episode. These normalized tonic episodes were divided into 100 intervals which represent the time series from the beginning (interval 1) to the end (interval 100). The mean ISI in each interval was calculated.

## Results

Nine Cav3.2 KO and 9 WT mice were used for the recording of VP and RT units under pentobarbital anesthesia. The probability of encountering at least one bursting neuron in recording tracks transverse the RT (histologically confirmed, Figure 3) was smaller for Cav3.2 KO mice (57.5% of the tracks) than WT mice (93.1%). In the following section, only well-isolated single units that had their receptive fields restricted within either the contralateral forelimb or hind limb will be reported (Table 1). By these criteria, 9477 bursts from 19 RT neurons in WT mice, 3815 bursts from 17 RT neurons in Cav3.2 KO mice, 9129 bursts from 47 VP neurons in WT mice, and 11,039 bursts from 29 VP neurons in Cav3.2 KO mice were analyzed. During the 10 min spontaneous activities recording period, RT units (n = 19) in Cav3.2 KO mice had an average 159.3 ± 42.7 burst events, which were significantly lower than those in WT (331.2 ± 62.4, 17 units).

**Table 1:** Comparison of burst unit %, burst rate and % of nociceptive RT and VP neurons in Cav3.2 KO and WT mice under pentobarbital and urethane anesthesia

	Pentobarbital				Urethane			
	**RT**		**VP**		**RT**		**VP**	

**Strain**	WT	KO	WT	KO	WT	KO	WT	KO
**Mouse N**	9	9	9	9	8	8	8	8
**% of burst^1^**	93.1	57.5	100	100	90	55	100	100
**Unit N**	17	19	29	47	8	7	9	8
**% nociceptive unit^2^**	65	42	10	4	50	57	0	0
**Spikes/s^3^**	8.6 ± 1.8^5^	5.1 ± 2.4*	0.8 ± 0.1	1.2 ± 0.1	21.2 ± 3.3	15 ± 2.5	2.3 ± 0.3	1.6 ± 0.4
**Bursts/s^4^**	0.53 ± 0.10	0.30 ± 0.06*	0.18 ± 0.03	0.27 ± 0.07*	1.42 ± 0.25	1.10 ± 0.17*	0.24 ± 0.09	0.40 ± 0.12

1, % of track with at least one typical RT or VP unit in tracks histologically identified

2, (nociceptive unit/total unit) × 100%

3, Average spike firing rate in 10 min recording period

4, Average burst rate in 10 min recording period

5, Mean ± SE

*: *P *< 0.05 by *t*-test of WT and KO comparison

The VP and RT burst firing patterns of the mouse were similar to those reported in cats and rats [2,19]. Figure 2A depicts the firing pattern of a RT unit recorded in a WT mouse. This unit showed several bursts in the beginning, followed by a long-lasting tonic spike tail. RT neurons in Cav3.2 KO mice also had these burst and long-lasting tonic firings (Figure 2D). In the expanded panels of Figure 2A and 2D, we found that 7 or 8 spikes could be discerned within a burst, and those burst showed acceleration and deceleration characteristics. The high-frequency bursts and tonic tail were identified as two dominant peaks in the inter-spike-interval (ISI) histogram (Figure 2C for WT and Figure 2F for KO mouse, respectively). Although qualitatively Cav3.2 KO RT neurons did not drastically differ from those of their WT controls, the ISI analysis of the RT unit in the KO mice showed less aggregation of the peaks (Figure 2C, F).

**Figure 4:**
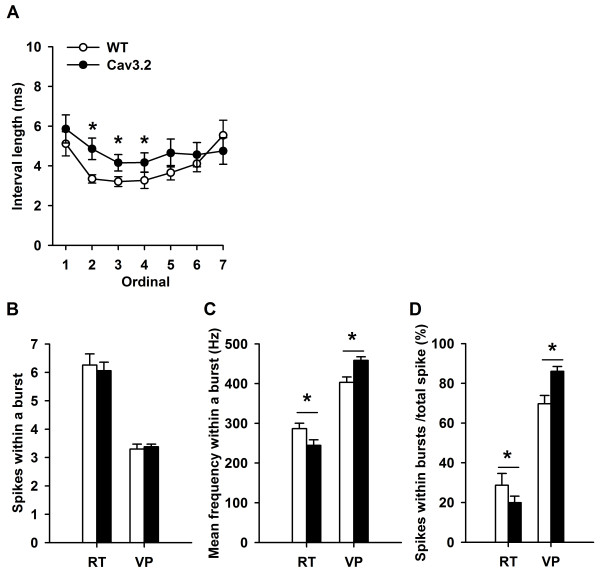
**Comparison of burst firing properties of RT and VP units in WT and Cav3.2 KO mice**. The accelerando-decelerando firing pattern (A), average number of spikes in a burst (B), mean firing frequency within a burst (C), and the percentage of spikes within bursts (D) were analyzed and compared between WT and KO mice. *: *p *< 0.05 between WT and KO groups.

We further analyzed the ISI pattern in a burst to examine whether the RT accelerando-decelerando signature changed in Cav3.2 KO mice. The first bursts in 27 episodes of 8 WT RT units and in 17 episodes of 7 Cav3.2 KO RT units were compared. Figure 4A shows that RT bursts were of longer interval lengths in Cav3.2 KO mice, particularly in the second (WT = 3.34 ± 0.2 ms, n = 27; KO = 4.86 ± 0.54 ms, n = 17), third (WT = 3.32 ± 0.2 ms; KO = 4.15 ± 0.48 ms), and fourth (WT = 3.27 ± 0.4 ms; KO = 4.17 ± 0.48 ms) intervals, which showed significant increase (One-way ANOVA followed by post-hoc pair-wise comparisons). Also of note is that in the ending period of a burst, a progressively increasing interval length (the decelerando part) was missing in Cav3.2 KO RT units (ordinals 5-7, Figure 4A).

The number of spikes within a burst (Figure 4B), the mean spike frequency within a burst (Figure 4C), and the percentage of spikes within bursts (Figure 4D) of RT and VP neurons were analyzed and compared. In additional to a lower average burst rate (Table 1), the RT units (n = 19) in Cav3.2 KO mice showed significant decreases in both the mean frequency within a burst (Figure 4C, WT = 286.6 ± 13.81Hz; KO = 244.6 ± 13.9 Hz) and the percentage of spikes within bursts (Figure 4D, WT = 28.7 ± 5.9%; KO = 19.9 ± 3.3%) than their WT counterparts (n = 17). In contrast, the VP units (n = 47) in Cav3.2 KO mice showed a significantly higher burst rate (Table 1), higher mean firing frequency within a burst (Figure 4C, WT = 403.1 ± 13.6 Hz; KO = 458.3 ± 9.2 Hz), and an increased percentage of spikes within bursts (Figure 4D, WT = 69.7 ± 4.1%; KO = 86 ± 2.4%), comparing to WT VP units (n = 29). No significant difference in the number of spikes within a burst in Cav3.2 KO mice was detected (Figure 4B, RT: WT = 6.3 ± 0.4; KO = 6.1 ± 0.3; VP: WT = 3.3 ± 0.2; KO = 3.4 ± 0.1).

To see whether the changes in the burst firing pattern are modality dependent, we divided the thalamic units into two modality types: light touch responsive (LT) and nociceptive based on their responsiveness to the pinching test (Figure 1). Classified this way, we had 11 nociceptive and 6 LT RT units, 3 nociceptive and 26 LT VP units in the WT control mice; and 8 nociceptive and 11 LT RT units, 2 nociceptive and 45 LT VP units in the Cav3.2 KO mice (Table 1). There seems to be a much lower ratio of pinch responsive VP neurons in the mouse thalamus in comparison to that of the rat [28]. The ratios of nociceptive VP units were even lower in the Cav3.2 KO mice than their WT control.

**Figure 5:**
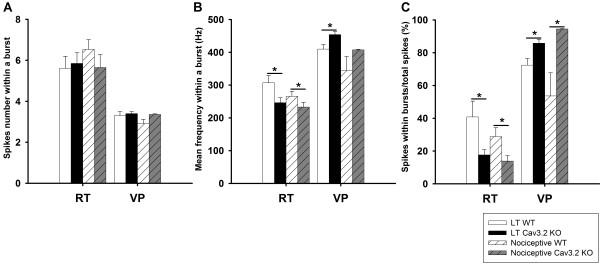
**Comparison of burst firing properties of LT and nociceptive RT and VP units in WT and Cav3.2 KO mice**. The white and black bars represent the values for the LT units and the slashed bars those of the nociceptive units. (A) Average number of spikes in a burst, (B) mean firing frequency within a burst, and (C) percentage of spikes within bursts were compared between the WT and the KO groups. *: *p *< 0.05 by *t*-test of WT and KO comparison

Figure 5 shows results of the burst analysis by modality. In the RT, both the LT and nociceptive units in Cav3.2 KO showed significantly reduced percentage of spike within bursts (Figure 5C). RT LT and nociceptive units showed the same tendency in their lower mean firing frequency within a burst (Figure 5B) in the Cav3.2 KO mice. Whereas in VP, the LT and nociceptive units showed significantly increased percentage of spike within bursts (Figure 5C), and LT and nociceptive units showed the same increasing tendency in their mean spike frequency within the burst (Figure 5B) in the Cav3.2 KO mice. No significant difference in the number of spikes within a burst in RT and VP (Figure 5A) neurons of Cav3.2 KO mice was detected.

In addition, we compared non-somatosensory units, this included 14 RT and 19 VP units in WT control, 14 RT and 10 VP units in Cav3.2 KO mice. These units were not responsive to any somatosensory stimuli tested. Similar pattern of change were observed between units in the KO versus those in the WT mice (data not shown). These indicate that Cav3.2 KO affects the thalamic firing properties in a general, modality-independent manner.

**Figure 6:**
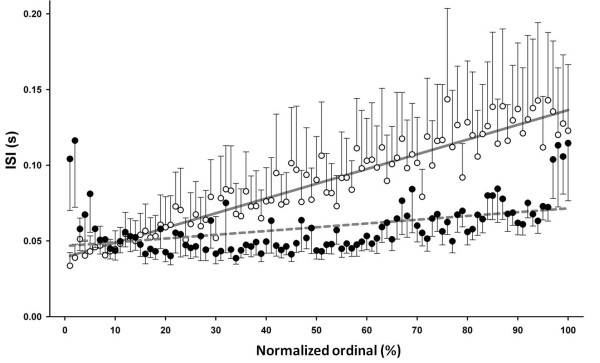
**Comparison of discharge pattern in the tonic firing episodes of RT units in WT and Cav3.2 KO mice**. In a tonic firing episode, RT unit of the WT mice (open circle) started at a shorter inter spike interval (ISI) and the interval gradually lengthened. X-axis represents normalized episode period (see text). The ISI of the WT RT units fitted well with the regression line (*r *= 0.91). In contrast, tonic firing of RT units of the Cav3.2 KO mice solid circles) showed less orderliness (*r *= 0.04). The gray lines represent linear regression fitting lines.

In addition to burst firing, we further compared the tonic firing of RT units of Cav3.2 KO mice. 253 RT tonic firing episodes in 13 WT units and 490 episodes in 17 KO units were analyzed. We found no statistical differences (*p *> 0.05 by *t*-test, WT 13 units, KO 17 units) in the following three characteristics of tonic firing between WT and Cav3.2 KO mice: tonic firing duration (WT = 10.4 ± 3.2 s; Cav3.2 KO = 15.4 ± 4.2 s); the number of spikes within a tonic firing train (WT = 187.3 ± 52.8; Cav3.2 KO = 373.6 ± 73.2), or the mean frequency within a train of tonic firing (WT = 24.4 ± 3.1 Hz; Cav3.2 KO = 30.6 ± 4.7 Hz). However, we note that the train of tonic firing of RT units usually starting at a higher frequency in the beginning and slowing down in the end of the train [2]. Whether this stereotypic tonic firing pattern is changed was analyzed in RT units of the Cav3.2 KO mice. We normalized tonic firing episodes of uneven lengths by dividing the full length into 100 intervals which represent the time series from the beginning (interval 1) to the end (interval 100). The results showed that RT units of the WT mouse had similar stereotypic tonic firing pattern as previously described in the cat [2], i.e., the ISI in the tonic firing episode began with shorter length (higher discharge frequency) that gradually increased (Figure 6 open circles). In contrast, tonic firing episodes of the Cav3.2 KO RT units did not have the graded increase in the interval length but was interspersed with irregular slower intervals, especially in the beginning and the end of the episodes (Figure 6 solid circles).

To examine whether the burst firing pattern changes in the Cav3.2 KO mice might have been caused by pentobarbital anesthesia, which is known to potentiate GABA receptor specifically, we performed another series of experiments using urethane anesthesia. In 8 Cav3.2 KO and 8 WT mice, there were still less tracks with RT bursts activities (55% in KO, compared with 90% in WT), and significantly lower average burst rate (1.1 Hz, 7 units in KO and 1.4 Hz, 8 units in WT, Table 1) in the Cav3.2 KO mice. Cav3.2 KO showed similar trend in increased burst firing in their VP units (0.4 Hz, 8 units in KO and 0.24 Hz, 9 units in WT) as in the pentobarbital anesthetized preparation. Also of note is that actual burst rates in every group were higher in the urethane anesthetized preparation than their counterparts in the pentobarbital anesthetized preparation (Table 1). These results indicate that pentobarbital anesthesia did not enhance burst firing pattern in the thalamic neurons and the observed burst firing pattern changes in Cav3.2 KO mice was not barbiturate-dependent.

## Discussion

The most important findings of the present study were: (1) More than 50% somatosensory RT neurons of the mice were nociceptive. (2) A decrease in the percentage of nociceptive RT and VP units of the Cav3.2 KO mice. (3) In Cav3.2 KO mice, fewer bursting RT neurons were found. In those spontaneous bursting RT neurons, they had fewer bursts, and the inter-spike intervals in the bursts were prolonged. (4) Bursting and tonic firing pattern of both nociceptive and tactile RT neurons in Cav3.2 KO mice changed significantly; (5) a lost of the regularity of the RT tonic firing in Cav3.2 KO mice;. And (6) in contrast, we found in Cav3.2 KO mice an increased firing frequency and an increased percentage of spikes in bursts of the VP neurons.

### Influences of Cav3.2 on RT burst firing

According to previous studies, different subtypes of T-channels show different regional expressions. In the rodent, Cav3.2 and Cav3.3 are co-expressed in RT neurons; on the other hand, VP neurons merely express Cav3.1 [18]. Among these three subtypes of T-channels, Cav3.1 and Cav3.2 have faster activation and inactivation channel kinetics than does Cav3.3 [17]. In this study, we found RT neurons in the Cav3.2 T-channel KO mice had fewer and slower bursts, and a weakening of the stereotypic accelerando-decelerando firing pattern. These changes correlate well with the disappearance of the fast Cav3.2 channel. On the other hand, the remaining slow Cav3.3 channel may still be able to induce a weaker Ca^2+ ^plateau, leading to rarer occurrence and slower burst firing.

One important firing signature of RT neurons is the long-lasting regular tonic spike train. It is thought that the third subtype of T-channel, Cav3.3, contributes to tonic firing by RT neurons. Cav3.3's current displays slow activation and inactivation kinetics that generate a sustained Ca^2+ ^current [32] and is highly correlated with intrinsic Ca^2+ ^oscillations [33]. Our data showed that after several burst firings, the RT neurons of WT mice showed a sustained regular single spike train with a progressive retardation of the rate of firing. However, Cav3.2 KO mice seemed to lose this regularity in their RT tonic tail (Figure 6), suggesting that Cav3.3 and Cav3.2 are both involved in modulating the long-lasting tonic firing of RT neurons.

### Influences of Cav3.2 on VP burst firing

Since VP neurons do not express Cav3.2, their firing properties were not supposed to change. However, we found VP neurons in pentobarbital or urethane anesthetized Cav3.2 KO mice had more bursts and burst faster. Anatomically, the RT nucleus, which is composed of GABAergic neurons, receives collaterals from thalamocortical and corticothalamic projections and provides major inhibitory inputs to the dorsal thalamus [26,27]. One possible explanation for the burst changes in VP neurons is that the tonic suppression by RT neurons was partially relieved in Cav3.2 KO mice. In Cav3.2 KO mice, RT neurons had fewer and weaker burst firings, and these de-inhibition changes allowed stronger and faster VP neuron bursts to occur. For example, the higher mean frequency within a burst of VP neurons in Cav3.2 KO mice (Figure 4C) can be explained by a reduced tonic GABAergic suppression by RT neurons [34]. VP neurons become slightly more excitable. When driving by excitatory inputs or by post-inhibitory rebounds, more T-channels can open, thus increasing the Ca^2+ ^influx. With faster Cav3.1 channels intact [14-16] and an increased Ca^2+ ^concentration, more voltage-gated Na^+ ^channels would open, thus shortening the spike intervals.

### Thalamic bursting and nociception

Recent studies suggested that burst firing of thalamic relays is highly correlated with nociception [12,35]. VP neurons of the Cav3.1 KO mice had greatly reduced burst firing, and showed enhanced behavioral responses to intraperitoneal acetic acid injection-induced visceral pain [12]. Thus, those authors proposed that nociceptive transmission in the thalamus may be suppressed by neuronal burst activities. In addition, Choi et al. reported that Cav3.2 KO mice showed attenuated pain responses to acute, thermal, and chemical pain tests [30]. Bourinet et al., using antisense targeted Cav3.2 to knock down Cav3.2 mRNA and protein expressions, showed distinct antinociceptive behaviors. The missing part here is whether Cav3.2 KO mice show increased bursts which are important in explaining the reduced nociception in Cav3.2 KO mice and supporting the antinociceptive property of thalamic bursts. Our results show that the burst frequency and mean frequency within each burst of VP neurons increased (Figure 4C, D) in Cav3.2 KO mice. Among these VP and RT neurons, some were nociceptive responsive, supporting the importance of thalamic bursts in nociceptive transmission.

### The RT input to VP

Rodent VP has few interneurons, it is composed mainly of thalamocortical (TC) neurons [23-25]. The TC neurons are a relatively homogenous population morphologically and chemically [27,36,37]. Nociceptive specific, wide dynamic range and low threshold neurons in the thalamus have similar morphology, membrane channels and receptors [26,38-41] and their spike waveforms did not show distinguishable features (from our unpublished data). It is the input and output of these neurons that distinguish their functional types. Examined this way, TC neurons in the VP, lateral geniculate nucleus, and medial geniculate nucleus share similar morphology and membrane properties. Therefore, it is not surprising that we found no functional specific change in the thalamus of the Cav3.2 KO mice. Why then are there pain behavior changes of these animals? Our explanation is that we were facing a morphologically homogenous population of VP TC neurons and another homogenous population of RT neurons. Cav3.2 KO targeted all RT neurons, but did not alter input-output connections in the thalamus. On the other hand, Cav3.2 genes are abundant in the dorsal root ganglia and spinal cord [31,42,43]. Manipulation of the Cav3.2 genes might have change the ascending input to the VP and RT. These changes in inputs were manifested in the decreased ratio of nociceptive thalamic neurons. Both changes in the bursting activity and the decreased ratio in nociceptive thalamic neurons contribute to the pain behavior changes of the Cav3.2 KO mice.

## Conclusions

Our results demonstrate the importance of Cav3.2 channels in the generation of burst firing and modulation of long-lasting tonic firing in RT neurons. Enhanced burst firings of VP neurons also provide support for thalamic burst firing being dynamically and reciprocally influenced by RT and VP interactions. The weakened RT gating and the enhanced VP bursting, coupled with decrease ratio of nociceptive neurons in the thalamus might be factors contributing to the changed pain behavior of the Cav3.2 KO mice.

## Competing interests

The authors declare that they have no competing interests.

## Authors' contributions

YCT and LYF conceived and designed the experiments. LYF and TML collected, analyzed, and interpreted data. YCT, LYF, TML, and CCC drafted the article and critically revised it. All authors read and approved the final version of the manuscript.
